# Association between bystander-initiated cardio-pulmonary resuscitation in pediatric out-of-hospital cardiac arrest and patient outcomes: Results from the French National Registry

**DOI:** 10.1016/j.resplu.2025.101105

**Published:** 2025-09-19

**Authors:** Marguerite Lockhart-Bouron, Valentine Baert, Stéphane Leteurtre, Matthieu Heidet, Hervé Hubert, Morgan Recher

**Affiliations:** a ULR 2694 - METRICS : Évaluation des technologies de santé et des pratiques médicales, Univ. Lille, CHU Lille, Lille, France; b French National Out-of-Hospital Cardiac Arrest Registry Research Group - Registre électronique des Arrêts Cardiaques, Lille, France; c Hôpital Henri Mondor and Université Paris-Est, Créteil, France - 1 rue Gustave Eiffel, 94000 Créteil, France

**Keywords:** Out-of-hospital cardiac arrest, Bystander-initiated cardiopulmonary resuscitation, Children, Return of spontaneous circulation, Propensity model

## Abstract

**Purpose:**

Effects of bystander cardiopulmonary resuscitation (CPR) on patient outcomes during pediatric out-of-hospital cardiac arrest (OHCA) remain to be fully elucidated. This study investigated bystander-initiated CPR effects on return of spontaneous circulation (ROSC) and survival at hospital admission in comparative pediatric population experiencing OHCA.

**Methods:**

Multicenter retrospective observational study conducted between January 2015 and December 2023 using the French National Cardiac Arrest Registry (RéAC) data. Pediatric patients (0–17 years) with OHCA were included. Patients who received bystander-initiated CPR were matched with those who did not using propensity score. Primary endpoints were ROSC and survival at hospital admission. Secondary endpoints were survival at D30 and favorable neurological outcomes (Cerebral Performance Category (CPC score of 1 or 2).

**Results:**

Of 2162 eligible pediatric patients, 1289 (59.6 %) received bystander-initiated CPR. After propensity score matching (n = 645 pairs), bystander-initiated CPR (vs without CPR by a bystander) was associated with improved ROSC (31.2 % vs 23.7 %; adjusted OR [AOR] 1.7, 95 % CI 1.1–1.9) and survival at hospital admission (28.7 % vs 19.8 %; AOR 1.7, 95 % CI 1.3–2.2). No significant difference was observed for survival at D30 (3.7 % vs 3.9 %; AOR 0.9, 95 % CI 0.5–1.7) or favorable neurological outcomes.

**Conclusions:**

In this nationwide French cohort, bystander-initiated CPR in pediatric OHCA was associated with improved ROSC and survival at hospital admission, but not with D30 survival or favorable neurological outcomes. These findings reinforce the importance of promoting bystander CPR, while highlighting the need for additional strategies to improve long-term outcomes in pediatric OHCA.

## Introduction

Pediatric out-of-hospital cardiac arrest (OHCA) represents a major public health concern in Europe.^1^ Despite advances in emergency systems, long-term survival in developed countries remains low, ranging from 6 to 12 %.^2^ The global incidence of pediatric OHCA is estimated at 6–10 per 100,000 person-years.^3^ In France, data from the French National OHCA Registry (RéAC) showed that, in one year period, children represented 1.8 % of 6918 OHCA cases, corresponding to an incidence of 5–10 per 100,000 person-years.^4^ Return of spontaneous circulation (ROSC) among pediatric cardiac arrest have improved over time, from 8.6 % to 32 %.^5^, ^6^, ^7^ Event survival is approximately 20 %.^5^ Survival to hospital discharge varies widely across studies, ranging from 0 to 26 %.^5^, ^8^ Factors associated with improved pediatric or adult survival include cardiac arrest due to medical causes, witnessed events, bystander CPR, rapid ROSC, and an initial shockable rhythm.^8^, ^9^, ^10^, ^11^, ^12^

The 2021 European Resuscitation Council guidelines and 2021 International Consensus on Cardiopulmonary Resuscitation recommend the initiation of early CPR in cases of pediatric cardiac arrest.^13^ This recommendation implies that bystanders should initiate CPR by any means, including phone instructions from the emergency team, while awaiting their arrival.^13^, ^14^

Previous studies on pediatric OHCA outcomes have shown mixed results regarding the impact of bystander CPR on survival. In a retrospective study conducted in England, bystander CPR for pediatric OHCA was associated with a higher ROSC rate but not with higher survival-to-hospital discharge rates.^15^ However, pediatric OHCA study results vary with respect to the effect of CPR by bystanders on survival to hospital discharge rates.^16^ To date, no study in France has compared the survival rates of OHCA in pediatric patients who received bystander-initiated CPR with those who did not.

Using the French National Cardiac Arrest Registry and including pediatric patients with OHCA, this study aimed to address this gap by comparing the rates of ROSC and survival status at hospital admission following pediatric OHCA with or without bystander-initiated CPR using propensity score matching. The secondary objective was to compare the survival status at day 30 (D30) or at hospital discharge (HD) in the same cohort.^17^

## Methods

### Study design

A registry-based multicenter retrospective observational study on pediatric OHCA was conducted between January 2015 and December 2023. Data were prospectively collected during the emergency medical service (EMS) intervention and stored in RéAC.^17^ In France, the prehospital emergency system comprises two tiers. First, the fire department quickly intervenes to provide basic life support (BLS). Second, mobile intensive care units (MICU) with physicians, nurses, and ambulance drivers provide patients with advanced life support (ALS).^18^ Since 2011, in cases of OHCA, all centers representing 311 MICU throughout Mainland France and French overseas territories have participated in RéAC by completing specific intervention modules that satisfy the requirements of French emergency medical services and the Utstein Resuscitation Registry.^19^

### Study data

Variables regarding patients’ characteristics were, age, sex, medical history; regarding the context of OHCA were the location (at home or other location), the presence of bystander at the time of OHCA (yes/no). Others data were assessed such as first documented rhythm, no-flow and low-flow times (time between first resuscitation gesture and ROSC or death), the initiation of ALS (including intubation and adrenaline injection), ROSC, vital status upon admission to hospital, and D30 follow-up data. All the definitions of collected data were based on the definition of the international Utstein Style template.^20^, ^21^

### Inclusion and exclusion criteria

Pediatric patients aged 0–17 years inclusive from the RéAC database. Exclusion criteria were patients with clear signs of death (rigor mortis, body findings: corresponding to bodies found dead with no indication of the time elapsed since CA), signs of prolonged OHCA (no flow > 60 min, with no flow corresponding to the absence of any form of resuscitation), end-of-life patients, spontaneous cardiac activity upon MICU arrival because of non-confirmation of actual CA, missing information about bystanders.

### Outcomes

This observational study compared pediatric OHCA patients who received bystander-initiated CPR and pediatric OHCA who did not receive bystander-initiated CPR. The primary outcomes were ROSC and survival to hospital admission. The secondary outcomes were the survival rate at D30 or hospital discharge after pediatric OHCA, and neurological outcomes were assessed using the Cerebral Performance Category (CPC) score for surviving patients at D30. A favorable neurological outcome was defined as a CPC score of 1–2.

### Statistical analysis

A propensity score was used to reduce the effect of confounding factors on between-group comparisons.^22^ Patients who received bystander-initiated CPR were matched with patients who did not received bystander-initiated CPR. The propensity score was estimated using logistic regression according to the presence of a bystander who started CPR as the dependent variable, and 17 covariates were selected from the model derived from the univariate analysis, including clinical and methodological relevance (covariates: age, respiratory medical history, other medical history, cause of cardiac arrest [medical, traumatic, or other causes], first aid provider BLS, initial cardiac rhythm [asystole, pulseless electrical activity (PEA), ventricular fibrillation, ventricular tachycardia], Mobile Medical Team (MMT) intubation, injection route [peripheral venous access, intraosseous, and no injection route], adrenaline dose, and low flow and no flow duration). Pediatric patients with OHCA who received bystander-initiated CPR were matched 1:1 to those who did not received bystander-initiated CPR based on the propensity score. The matching distance used was the nearest neighbor with a caliper of 0.2 X of the propensity score standard deviation.^23^, ^24^ To assess the matching quality, we assessed the absolute standardized differences (ASD) for covariates before and after matching. An ASD > 0.1 was considered an indication of a meaningful imbalance in the baseline covariates. Conditional logistic regression was used to estimate the adjusted OR (AOR). All statistical analyses were performed using SPSS software (v.25.0; IBM, Armonk, NY, USA).

The normality of the quantitative variable distributions was assessed using the Kolmogorov-Smirnov test. Quantitative variables are described as medians [first quartile, third quartile] or mean [standard deviation]. Qualitative variables were described as frequency. Bivariate analyses were performed using the Pearson chi-square test for categorical variables and the Mann–Whitney *U* test for continuous variables. The odds ratios (OR) and confidence intervals (CI) were calculated for D30 survival, ROSC, survival at hospital admission, and neurological outcomes at hospital discharge for both groups. The level of significance was set at p < 0.05. The absolute reduction risk (ARR) with CI and the number needed to treat (NNT) was calculated for survival at hospital admission, ROSC and D30 survival.

## Results

### Study population

**Fig. 1:**
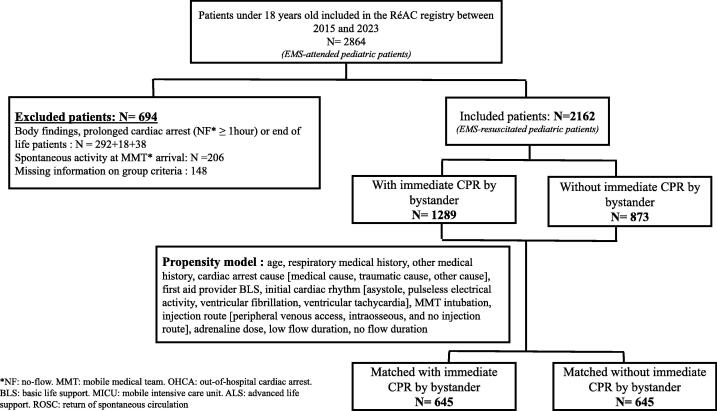
Study flow-chart. *NF: no-flow. MMT: mobile medical team. OHCA: out-of-hospital cardiac arrest. BLS: basic life support. MICU: mobile intensive care unit. ALS: advanced life support. ROSC: return of spontaneous circulation.

During the study period, 2864 pediatric patients were included in the RéAC registry. After applying the exclusion criteria, 2162 pediatric patients were included in the analysis (Fig. 1). Overall, 1289 (59.6 %) pediatric patients with OHCA received bystander-initiated CPR, and 873 (40.3 %) pediatric patients experienced OHCA with no bystander-initiated CPR (Table 1). No differences in age, sex, location (at home or outside the home), or time of day (day or night) of the OHCA events were observed between the two groups. Previous respiratory or other medical history was more frequent in the group that received bystander-initiated CPR (5.5 % vs. 3.6 %, p = 0.03; 24.4 % vs. 20.6 %, p = 0.04, respectively). Traumatic OHCA events were more frequent in the group without a bystander-initiated CPR (27.8 % vs. 16.2 %, p < 0.001), whereas medical OHCA and other causes (burn, asphyxia, electrocution, and drowning) were more frequent in the group receiving bystander-initiated CPR (61.4 % vs. 54.6 %, p < 0.001; 22.3 % vs. 17.5 %, p < 0.001). The initial cardiac rhythm differed between the groups, with asystole being more frequent in patients with OHCA without a bystander (88.1 % vs. 93.7 %, p < 0.001), whereas pulseless electrical activity and ventricular fibrillation were more frequent among patients receiving bystander-initiated CPR (6.8 % vs. 4.5 %, p < 0.001; 5.1 % vs. 1.8 %, p < 0.001). The proportion of patients requiring intubation was higher in the bystander-initiated CPR OHCA group (91 % vs. 78 %, p < 0.001). The injection route differed between the two groups, with a higher proportion of intraosseous and peripheral intravenous access in the bystander-initiated CPR group. No difference was observed in the time from call to first aid provider or MICU arrival. The no-flow duration for patients with bystander-initiated CPR for OHCA was 5 min [0;13] compared with 11 min [6;17] for patients who did not receive bystander-initiated CPR (p < 0.001). The low-flow duration for patients with bystander-initiated CPR for OHCA 40 min [26;55] compared with 35 min [20;49] for patients who did not receive bystander-initiated CPR (p < 0.001). All group differences in the variables are listed in Table 1.

**Table 1:** Patients population characteristics.

	Overall N = 2,162	Patients with immediate CPR by bystander N = 1289	Patients without immediate CPR by bystander N = 873	p
**Age (years) M [Q1; Q3]**		4 [0;13]	5 [0;14]	0.306

Time of the day*(1552)				0.063
Night (%)	522 (33.6)	302 (31.9)	220 (36.4)	
Day (%)	1030 (66.4)	646 (68.1)	384 (63.6)	

**Sex: male, n (%)**	1330 (61.5)	789 (61.2)	541 (62.0)	0.674

**CA location, home n (%)**	1306 (65.5)	791 (66.9)	515 (63.3)	0.099

**Medical history**				
Cardiac, n (%)	125 (5.8)	74 (5.7)	51 (5.8)	0.921
Respiratory, n (%)	102 (4.7)	71 (5.5)	31 (3.6)	0.035
Other, n (%)	494 (22.8)	314 (24.4)	180 (20.6)	0.042
None, n (%)	808 (37.4)	485 (37.6)	323 (37)	0.767

**Cause of cardiac arrest**				<0.001
Medical, n (%)	1269 (58.7)	792 (61.4)	477 (54.6)	
Traumatic, n (%)	452 (20.9)	209 (16.2)	243 (27.8)	
Other, n (%)	441 (20.4)	288 (22.3)	153 (17.5)	

**Basic Life Support**				
Bystander’s presence, n (%)	1243 (57.5)	812 (63)	431 (49.4)	<0.001
First aid provider BLS, n (%)	1873 (86.6)	1174 (91.1)	699 (80.1)	<0.001

**AED Shock (before MMT arrival), n (%)**	138 (6.4)	104 (8.1)	34 (3.9)	<0.001

**Initial cardiac rhythm at MMT arrival, n (%)**				<0.001
Asystole	1906 (90.3)	1116 (88.1)	790 (93.7)	
PEA	124 (5.9)	86 (6.8)	38 (4.5)	
VF/pulseless VT	80 (3.8)	65 (5.1)	15 (1.8)	

**MMT intubation, n(%)**	1847 (85.4)	1166 (90.5)	681 (78)	<0.001

**Injection route**				
IO	989 (45.7)	632 (49)	357 (40.9)	<0.001
PIV	1025 (47.4)	646 (50.1)	379 (43.4)	0.002
CIV	25 (1.2)	19 (1.5)	6 (0.7)	0.093
Endotracheal	57 (2.6)	31 (2.4)	26 (3)	0.414
None	229 (10.6)	65 (5)	164 (18.8)	<0.001

**Adrenaline**	1830 (84.7)	1151 (89.4)	679 (77.9)	<0.001

**Adrenaline dose M [Q1; Q3]**		1.6 [0.5;6]	2 [0.5;6]	<0.001

**Care timing (minutes)**				
Call (T0) to first aid provider arrival M [Q1; Q3]		10 [6;13]	9 [5;13]	0.344
Call (T0) to MICU arrival M [Q1; Q3]		17 [12;24]	18 [12;25]	0.351
Call (T0) to ROSC or death M [Q1; Q3]		48 [35;62]	46 [34;61]	0.229

**No-flow duration M [Q1; Q3]**		5 [0;13]	11 [6;17]	<0.001

**Low-flow duration M [Q1; Q3]**		40 [26;55]	35 [20;49]	<0.001

**Survival**				
ROSC	514 (23.8)	350 (27.2)	164 (18.8)	<0.001
Survival at hospital admission	566 (26.2)	404 (31.3)	162 (18.6)	<0.001
D30 survival	94 (4.3)	68 (5.3)	26 (3)	0.01
If D30 alive, CPC 1–2	53 (2.5)	39 (3.1)	14 (1.6)	0.03

* (xx) patients, missing data.

### Non-adjusted comparison of bystander-initiated CPR vs no bystander-initiated CPR OHCA

**Fig. 2:**
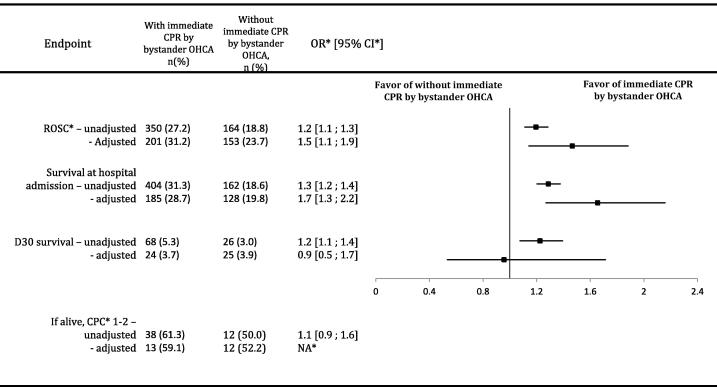
Survival at D0 and D30 and ROSC in non-adjusted and adjusted populations. *OHCA: out-of-hospital cardiac arrest. OR: odds ratios. CI: confident interval. ROSC: return of spontaneous circulation. NA: not applicable. ARR: absolute risk ratio.

Patients in the bystander-initiated CPR OHCA group compared with the no bystander-initiated CPR OHCA group (Fig. 2) had a higher survival at hospital admission rate (31.3 % vs. 18.6 %, OR 1.3, 95 % CI 1.2–1.4), a higher ROSC rate (27.2 % vs. 18.8 %, OR 1.2, 95 % CI 1.1–1.3), and a higher D30 survival rate (5.3 % vs. 3.0 %, OR 1.2, 95 % CI 1.1–1.4). No difference in neurological outcome was observed between the two groups. If alive, 38 (61.3 %) patients had good neurological outcomes (CPC 1 or 2) in OHCA cases receiving bystander-initiated CPR and 12 patients (50 %) in OHCA cases with no bystander-initiated CPR (OR 1.1, CI 95 % 0.9–1.6).

### Adjusted comparison of bystander-initiated CPR for OHCA by a bystander versus no bystander-initiated CPR

A total of 645 matched patients were enrolled in the study (Fig. 2 and Supplementary Fig. 1) according to propensity score matching with 17 covariates (Table 2). ROSC and survival at hospital admission rates were higher in the group with bystander-initiated CPR (31.2 % vs. 23.7 %, AOR 1.5, 95 % CI 1.1–1.9; 28.7 % vs. 19.8 %, AOR 1.7, 95 % CI 0.46–0.78). Pediatric patient with OHCA who received bystander-initiated CPR were 7.4 % more likely to achieve ROSC (ARR −7.4 %, 95 % CI 12 %– −2.5 %) with a NNT of 13 (95 % CI 39–8). Bystander-initiated CPR patients were 8.8 % more likely to survive at hospital admission (ARR −8.8 %, 95 % CI −13 % – −4.1 %) with a NNT of 11 (95 % CI 24–7). There was no difference in survival rate at D30 (3.7 % vs. 3.9 %, AOR 0.9, 95 % CI 0.5–1.7). Only 13 (59.1 %) patients in the bystander-initiated CPR group had good neurological outcomes with CPC score of 1 or 2 and only 12 patients (52.2 %) in no bystander-initiated CPR group, OR could not be calculated between both groups due to poor number of survivors with good neurological outcomes.

**Table 2:** Covariates after matching (N = 645).

	Bystander-initiated CPR	No bystander-initiated CPR	ASD
Age, mean (SD)	6.4 (6.4)	6.5 (6.6)	0.01
Respiratory medical history, n (%)	28 (4.3)	29 (4.5)	0.009
Other medical history, n (%)	122 (18.9)	121 (18.8)	0.002
CA* from medical causes, n (%)	389 (60.3)	356 (55.2)	0.1
CA*from traumatic causes, n (%)	122 (18.9)	163 (25.3)	0.1
CA*from other causes, n (%)	134 (20.8)	126 (19.5)	0.03
First aid provider BLS*, n (%)	569 (88.2)	567 (87.9)	0.009
Initial cardiac rhythm: Asystole, n (%)	610 (94.6)	599 (92.9)	0.07
Initial cardiac rhythm: Pulsless electric activity, n (%)	27 (4.2)	33 (5.1)	0.04
Initial cardiac rhythm: ventricular fibrillation or tachycardia, n (%)	8 (1.2)	13 (2)	0.06
MMT* intubation, n (%)	607 (94.1)	609 (94.4)	0.01
Injection route: intra-venous access, n (%)	350 (54.3)	338 (52.4)	0.03
Injection route: intra-osseous access, n (%)	313 (48.5)	322 (49.9)	0.02
Injection route: no injection route, n (%)	8 (1.2)	11 (1.7)	0.04
Adrenaline dose, mean (SD)	3.7 (4.5)	3.6 (4.2)	0.03
No flow duration, mean (SD)	11.1 (10.5)	12.2 (8.8)	0.009
Low flow duration, mean (SD)	40.3 (22.8)	40.9 (20.7)	0.02

* CA: cardiac arrest, BLS: Basic Life Support, MMT: Mobile Medical Team.

## Discussion

This is the first French multicenter study using national database to compare ROSC and survival rates in pediatric patients experiencing OHCA who received bystander-initiated CPR and those who did not receive bystander-initiated CPR using a matching methodology. Pediatric patients experiencing OHCA accompanied bystander-initiated CPR were characterized more often by previous respiratory or other medical history, and more frequent incidence of OHCA from medical or other causes. In the adjusted comparisons, the ROSC and survival at hospital admission rates were higher in the group with bystander-initiated CPR. The D30 or hospital discharge survival rates were similar across groups.

In the pediatric population, the average rate of bystanders providing CPR intervention was 47 % in the USA, 69.6 % in England, 55 % in Germany, and 75 % in North European countries.^15^, ^16^, ^25^, ^26^ An increase in pediatric OHCA cases identified in the call and receiving bystander-CPR has been reported in recent years,^10^ it may be attributed to the increase in CPR training programs for the general population.^11^

In our study, pediatric patients with OHCA who received bystander-initiated CPR had a higher rate of ROSC and survival at hospital admission rate. This finding is consistent with the results of previous retrospective studies.^15^, ^26^ Katzenschlager et al. reported an even higher ROSC upon hospital admission when bystander CPR was performed with ventilation.^26^ Children with pre-existing conditions have a higher risk of OHCA.^27^ Our pediatric population with OHCA and bystander-initiated CPR more often had a history of respiratory or other medical conditions. The proportion of pediatric patients experiencing OHCA due to respiratory causes was previously reported to be up to 30 %.^26^ Furthermore, pediatric OHCAs caused by respiratory arrest accounted for 29 % of cases in children and up to 36 % in infants.^7^ In a study involving the adult French population, the availability of a bystander who performed CPR after OHCA was associated with a higher D30 survival rate.^11^ Similar findings were observed in the pediatric population with non-adjusted comparisons. However, these results were not confirmed by the adjusted comparison. A possible explanation could be that the adult population mostly requires thoracic compression and defibrillation, as the etiology is mostly cardiac.^28^ This may differ in pediatric populations, in which respiratory causes are mostly involved.^1^, ^3^ The 2021 European Resuscitation Council did not change its recommendations concerning the need for resuscitation breathing for pediatric populations, and some studies reported a higher long-term survival rate following conventional CPR, including rescue breathing, than that for chest-compression CPR interventions alone.^13^, ^26^, ^29^

Bystander-initiated CPR were not associated with improved survival on D30 or survival-to-hospital discharge, as described in previous studies.^15^, ^27^ No predictors of survival until hospital discharge were observed. Banerjee et al.^12^ reported shorter MICU arrival times.^12^ Additionally, predictors of ROSC and survival at hospital admission include bystander treatment before MICU arrival.^12^ A short post-ROSC period might be crucial for improving the D30 survival rate by limiting persistent low blood pressure, bradycardia, low urine output, hypothermia, and persistent hypoxemia.^13^, ^27^ Management of post-ROSC varies from one center to another and might greatly influence the survival rate at D30 or at hospital discharge, which could explain the lack of difference in the D30 survival rate between groups receiving bystander-initiated CPR and those without bystander-initiated CPR.^13^ An explanation for the low D30 survival rate in our population might be due for part to the inclusion of traumatic pediatric OHCA but the use of propensity matching method permits to have comparable group with as many traumatic pediatric OHCA in both groups. Also, for children found in asystole or with pulseless electrical activity studies reporting 0 to 8 % survival rate at hospital discharge, which is close to our study. Finally, we did not separate infants, children or adolescents which could also explain the low D30 survival rate.^3^ Due to the low number of survivors, we did not find differences in neurological outcomes between the groups, although this has been reported in previous studies with dispatcher-assisted bystander CPR, especially with conventional CPR.^30^

Study periods included COVID-19 pandemic, but it might not interfere as in pediatric population in France, no deaths were directly linked to COVID-19.^31^ Also, there were no difference in care of pediatric OHCA during COVID period which enabled not to adjust for year.^32^

Our study had many strengths. This is the first study to compare the impact of the presence or absence of bystander-initiated CPR in pediatric patients with OHCA in comparable populations using propensity score. This study used data from a nationwide registry of pediatric patients with OHCA. This is the first French pediatric study based on data prospectively collected that established a positive effect of bystander-initiated CPR in pediatric OHCA on ROSC and survival at hospital admission, even if no difference was found for the adjusted population in terms of the survival rate at hospital discharge.

Although RéAC is a nationwide registry, during the study period, not all French emergency medical services registered complete data, with RéAC including 94 % of French MICU.^17^ Our findings are predominantly applicable to France, given the distinct characteristics of the French system that differentiate it from other countries, even though it examines the effects of bystander response in a context of pediatric cardiac arrest not different from one country to another. Furthermore, the precise characteristics of bystander interventions providing CPR are difficult to obtain, especially the inclusion of ventilation with chest compression, which could influence survival and the quality of post-cardiac arrest care. Propensity matched approach is innovative and allows us to obtain comparable groups, particularly concerning cardiac arrest causes (traumatic vs medical). Although it reduces number of patients per group due to impossible matching. An inherent limitation of our study is that we relied on registry data. As such, some variables are not mandatory and may be subject to missing data. For example, it would have been relevant to include the bystander's status in the construction of the propensity score; however, the proportion of missing data for this variable was too high. Nonetheless, the propensity score was developed based on both methodological and clinically relevant criteria, which allowed for the creation of a substantial number of matched patient pairs. Finally, data related to pediatric patients from January 2011 to December 2014 were not collected, as improvements in data collection since 2015 have resulted in more complete data.

## Conclusion

In two comparable French pediatric populations, the presence of active bystanders providing CPR in patients with OHCA was associated with a higher rate of survival at hospital admission and ROSC but not with a higher rate of survival at D30 or survival at discharge. Owing to the limited number of survivors in the sample population, no conclusions could be drawn regarding neurological outcomes. These findings reinforce the importance of promoting bystander CPR, while highlighting the need for additional strategies to improve long-term outcomes in pediatric OHCA.

## Authors contributions

All authors contributed to collect the study data, revised the manuscript for important intellectual content, and read and approved the final version of the manuscript.

MLB and MR conceived and designed the study; analysed and interpreted the data; drafted the manuscript, and revised the manuscript for important intellectual content.

## Consent for publication

Not applicable.

## CRediT authorship contribution statement

**Marguerite Lockhart-Bouron:** Writing – original draft, Visualization, Methodology, Formal analysis, Conceptualization. **Valentine Baert:** Methodology, Formal analysis. **Stéphane Leteurtre:** Writing – review & editing. **Matthieu Heidet:** Writing – review & editing. **Hervé Hubert:** Writing – review & editing. **Morgan Recher:** Writing – review & editing, Methodology, Conceptualization.

## Ethics approval

The study was approved by the French advisory committee on information processing in health research and by the French National Data Protection Commission (authorisation number 910946). This study was approved as a medical assessment registry without the requirement for patient consent.

## Funding

This research did not receive any specific grant from funding agencies in the public, commercial, or not-for-profit sectors.

## Declaration of competing interest

The authors declare that they have no known competing financial interests or personal relationships that could have appeared to influence the work reported in this article.

## References

[1] Kiguchi T., Okubo M., Nishiyama C. (2020). Out-of-hospital cardiac arrest across the World: first report from the International Liaison Committee on Resuscitation (ILCOR). Resuscitation.

[2] Berdowski J., Berg R.A., Tijssen J.G.P., Koster R.W. (2010). Global incidences of out-of-hospital cardiac arrest and survival rates: systematic review of 67 prospective studies. Resuscitation.

[3] Rossano J.W., Naim M.Y., Nadkarni V.M., Berg R.A., Da Cruz E.M., Ivy D., Jaggers J. (2014). Pediatric and congenital cardiology, cardiac surgery and intensive care.

[4] Luc G., Baert V., Escutnaire J. (2019). Epidemiology of out-of-hospital cardiac arrest: a French national incidence and mid-term survival rate study. Anaesth Crit Care Pain Med.

[5] Alqudah Z., Nehme Z., Williams B., Oteir A., Bernard S., Smith K. (2019). A descriptive analysis of the epidemiology and management of paediatric traumatic out-of-hospital cardiac arrest. Resuscitation.

[6] Inoue M., Tohira H., Williams T. (2017). Incidence, characteristics and survival outcomes of out-of-hospital cardiac arrest in children and adolescents between 1997 and 2014 in Perth, Western Australia: trends of paediatric out-of-hospital cardiac arrest. Emerg Med Australas.

[7] Nitta M., Iwami T., Kitamura T. (2011). Age-specific differences in outcomes after out-of-hospital cardiac arrests. Pediatrics.

[8] Alqudah Z., Nehme Z., Alrawashdeh A., Williams B., Oteir A., Smith K. (2020). Paediatric traumatic out-of-hospital cardiac arrest: a systematic review and meta-analysis. Resuscitation.

[9] Lockhart-Bouron M., Baert V., Leteurtre S., Hubert H., Recher M. (2023). Association between out-of-hospital cardiac arrest and survival in paediatric traumatic population: results from the French national registry. Eur J Emerg Med.

[10] Nehme Z., Namachivayam S., Smith K., Forrest A., Butt W. (2018). Trends in the incidence and outcome of paediatric out-of-hospital cardiac arrest: a 17-year observational study. Resuscitation.

[11] Lafrance M., Recher M., Javaudin F. (2023). Bystander basic life support and survival after out-of-hospital cardiac arrest: a propensity score matching analysis. Am J Emerg Med.

[12] Banerjee P., Ganti L., Stead T.G., Vera A.E., Vittone R., Pepe P.E. (2021). Every one-minute delay in EMS on-scene resuscitation after out-of-hospital pediatric cardiac arrest lowers ROSC by 5. Resusc plus.

[13] Van de Voorde P., Turner N.M., Djakow J. (2021). European Resuscitation Council guidelines 2021: paediatric life support. Resuscitation.

[14] Wyckoff M.H., Singletary E.M., Soar J. (2021). International Consensus on Cardiopulmonary Resuscitation and Emergency Cardiovascular Care Science With Treatment Recommendations: summary from the basic life support; advanced life support; neonatal life support; education, implementation, and teams; first Aid task forces; and the COVID-19 working group. Circulation.

[15] Albargi H., Mallett S., Berhane S. (2022). Bystander cardiopulmonary resuscitation for paediatric out-of-hospital cardiac arrest in England: an observational registry cohort study. Resuscitation.

[16] Naim M.Y., Burke R.V., McNally B.F. (2017). Association of bystander cardiopulmonary resuscitation with overall and neurologically favorable survival after pediatric out-of-hospital cardiac arrest in the united states: a report from the cardiac arrest registry to enhance survival surveillance registry. JAMA Pediatr.

[17] Hubert H., Tazarourte K., Wiel E. (2014). Rationale, methodology, implementation, and first results of the French out-of-hospital cardiac arrest registry. Prehosp Emerg Care.

[18] Javaudin F., Penverne Y., Montassier E. (2020). Organisation of prehospital care: the French experience. Eur J Emerg Med.

[19] Perkins G.D., Jacobs I.G., Nadkarni V.M. (2015). Cardiac arrest and cardiopulmonary resuscitation outcome reports: update of the Utstein resuscitation registry templates for out-of-hospital cardiac arrest. Resuscitation.

[20] Idris A.H., Bierens J.J.L.M., Perkins G.D. (2017). 2015 revised utstein-style recommended guidelines for uniform reporting of data from drowning-related resuscitation: an ILCOR advisory statement. Circ: Cardiovasc Qual Outcomes.

[21] Bray J.E., Grasner J.-T., Nolan J.P. (2024). Cardiac arrest and cardiopulmonary resuscitation outcome reports: 2024 update of the Utstein out-of-hospital cardiac arrest registry template. Circulation.

[22] Austin P.C. (2011). An introduction to propensity score methods for reducing the effects of confounding in observational studies. Multivar Behav Res.

[23] Austin P.C. (2011). Optimal caliper widths for propensity‐score matching when estimating differences in means and differences in proportions in observational studies. Pharmaceut Statist.

[24] Imbens G.W. (2004). Nonparametric estimation of average treatment effects under exogeneity: a review. Rev Econ Stat.

[25] Gelberg J., Strömsöe A., Hollenberg J. (2015). Improving survival and neurologic function for younger age groups after out-of-hospital cardiac arrest in Sweden: a 20-year comparison. Pediatr Crit Care Med.

[26] Katzenschlager S., Kelpanides I.K., Ristau P. (2023). Out-of-hospital cardiac arrest in children: an epidemiological study based on the German Resuscitation Registry identifying modifiable factors for return of spontaneous circulation. Crit Care.

[27] Lee J., Yang W.-C., Lee E.-P. (2019). Clinical survey and predictors of outcomes of pediatric out-of-hospital cardiac arrest admitted to the emergency department. Sci Rep.

[28] Yan S., Gan Y., Jiang N. (2020). The global survival rate among adult out-of-hospital cardiac arrest patients who received cardiopulmonary resuscitation: a systematic review and meta-analysis. Crit Care.

[29] Kitamura T., Iwami T., Kawamura T. (2010). Conventional and chest-compression-only cardiopulmonary resuscitation by bystanders for children who have out-of-hospital cardiac arrests: a prospective, nationwide, population-based cohort study. Lancet.

[30] Goto Y., Maeda T., Goto Y. (2014). Impact of dispatcher‐assisted bystander cardiopulmonary resuscitation on neurological outcomes in children with out‐of‐hospital cardiac arrests: a prospective, nationwide, population‐based cohort study. J Am Heart Assoc.

[31] Lockhart-Bouron M., Vanel N., Levy M. (2024). Severe acute respiratory syndrome coronavirus-2-related and imputable deaths in children: results from the French pediatric national registry. World J Pediatr.

[32] Recher M., Baert V., Leteurtre S., Hubert H. (2020). Consequences of coronavirus disease outbreak on paediatric out-of-hospital cardiac arrest in France. Resuscitation.

