# Supplementary material for: Association between bystander-initiated cardio-pulmonary resuscitation in pediatric out-of-hospital cardiac arrest and patient outcomes: Results from the French National Registry
Source: Resusc Plus. 2025 Sep 19;26:101105. doi: 10.1016/j.resplu.2025.101105 (PMC12513290; doi:10.1016/j.resplu.2025.101105)
Supplement: Supplementary Data 1 [file mmc1.pdf]

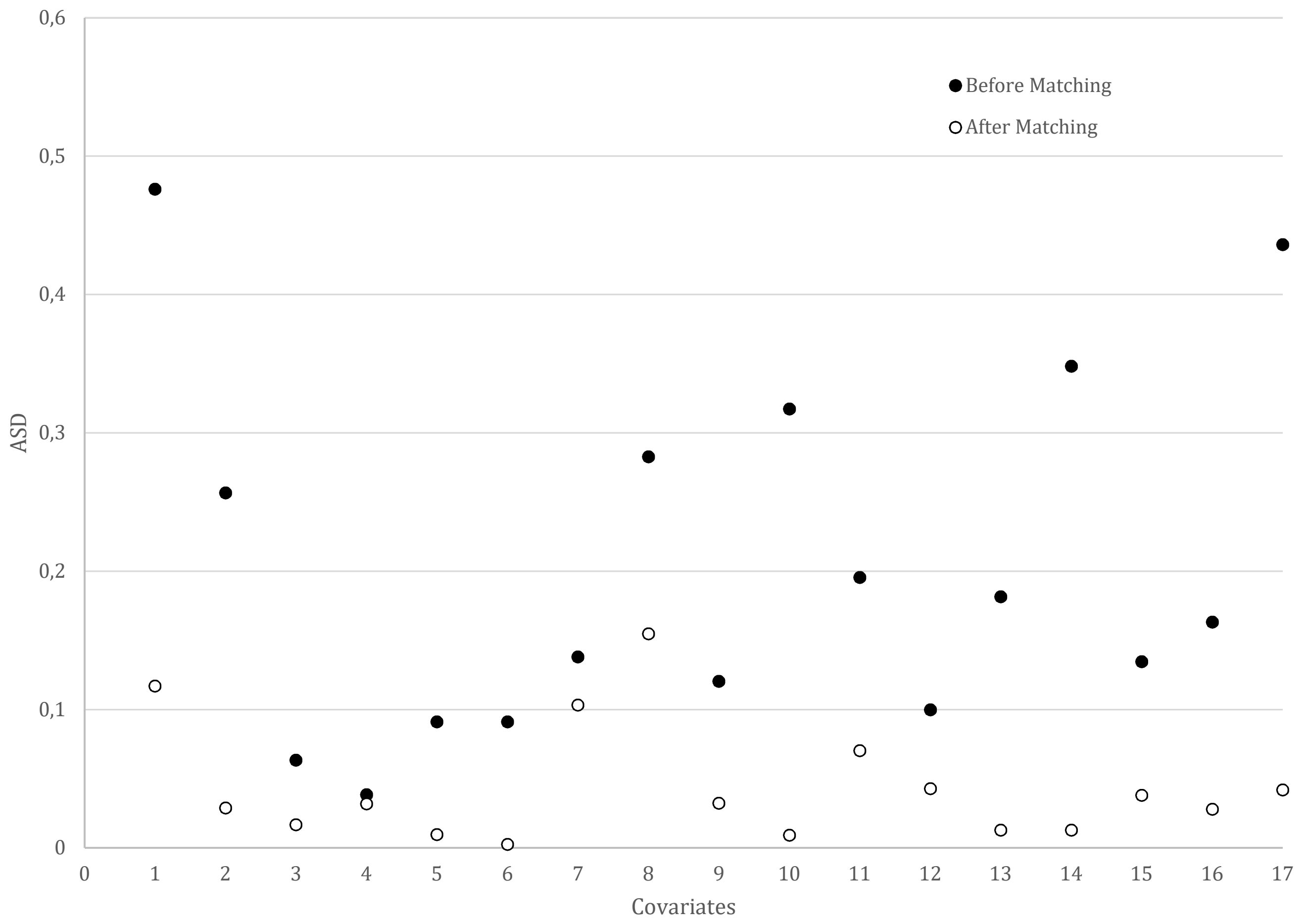

Supplementary Figure 1. Absolute standardised differences before and after matching

1. Now flow, 2. Low flow, 3. Age, 4. Adrenaline dose, 5. Respiratory medical history, 6. Other medical history, 7. Medical cause, 8. Traumatic cause, 9. Other cause, 10. First aid BLS, 11. Asystole, 12. Pulsless Electrical Activity, 13. Ventricular Fibrillation/Ventricular Tachycardia, 14. Intubation, 15. Peripheral Venous Access, 16. Intra-Osseous Access, 17. No injection route
